# Appetitive Traits and Dietary Patterns in Mexican Children Aged 12 to 36 Months

**DOI:** 10.3390/nu17111814

**Published:** 2025-05-27

**Authors:** Astrid S. Gil-Barrera, Claudia Hunot-Alexander, Clío Chávez-Palencia, Jocelyn González-Toribio, Erika Casillas-Toral, D. Citlalli Álvarez-Zaragoza, Alfredo Larrosa-Haro, Edgar Vásquez-Garibay

**Affiliations:** 1 Instituto de Nutrición Humana, Centro Universitario de Ciencias de la Salud, Universidad de Guadalajara, Salvador Quevedo y Zubieta #750, Edificio Anexo al Hospital Civil “Dr. Juan I. Menchaca”, Piso 3, Guadalajara 44340, Jalisco, Mexico; asgilb@unal.edu.co (A.S.G.-B.); citlalli.nutricion@hotmail.com (D.C.Á.-Z.); alfredo.larrosa@academicos.udg.mx (A.L.-H.); edgar.vgaribay@academicos.udg.mx (E.V.-G.); 2 División de Ciencias de la Salud, Centro Universitario de Tonalá, Universidad de Guadalajara, Av. Nuevo Periférico No. 555 Ejido San José Tateposco, Tonalá 45425, Jalisco, Mexico; 3 Casa de la Amistad para Niños con Cáncer, I.A.P. Aldama 2, San Juan Tepepan, Xochimilco, Ciudad de México 16020, Mexico; erika.casillastoral@gmail.com

**Keywords:** appetitive traits, dietary patterns, appetite, feeding practices, toddlers

## Abstract

**Background/Objectives:** Appetitive traits may contribute to early feeding challenges by shaping children’s emerging dietary patterns. While food approach traits have been linked to excess weight, their role in influencing food type and quality during toddlerhood remains underexplored. This study aimed to examine associations between appetitive traits and dietary patterns in children aged 12 to 36 months. **Methods:** This cross-sectional study collected data from a university hospital and the metropolitan area of Guadalajara, Mexico. A survey was conducted through direct interviews with primary caregivers, which included the Child Eating Behavior Questionnaire for Toddlers (CEBQ-T) and a qualitative food group frequency questionnaire. Dietary patterns were determined using principal component analysis. Statistical analyses were performed to identify associations between six appetitive traits and dietary patterns. **Results:** Three dietary patterns were identified: “Processed”, “Healthy” and “Dietary Transition/Modern Mexican”. Higher scores for Food Responsiveness and Emotional Overeating traits were associated with greater adherence to the Processed dietary pattern. Increased scores in Enjoyment of Food were associated with higher adherence to a Healthy dietary pattern. Children with higher scores in Food Fussiness exhibited lower adherence to the Healthy dietary pattern and were more likely to follow a Dietary Transition/Modern Mexican pattern. Between 12 and 36 months of age, appetitive traits may influence the development of more or less healthy dietary patterns. **Conclusions:** These findings underscore the importance of early identification of appetite-related behavioral tendencies as part of a broader understanding of feeding challenges in early childhood.

## 1. Introduction

Establishing healthy eating patterns from birth to five years of age and maintaining them thereafter can contribute to the prevention of chronic diseases, optimal physical and cognitive development, and overall health [1,2,3]. From birth to three years of age, the brain undergoes rapid development, making adequate nutrition, protection, and positive stimulation essential [4]. Between one and three years of age, children begin to develop social skills that lead them to imitate the eating behaviors of those around them, which likely results in the adoption of their household’s dietary patterns through observational learning [5,6].

Childhood overweight and obesity remain pressing public health challenges in Mexico, with a prevalence of 6.7% among children under five, according to the 2023 Continuous National Health and Nutrition Survey Continua (ENSANUT CONTINUA 2023) [7]. The same survey highlights dietary patterns: among children aged one to four years, 80.6% consume sugar-sweetened beverages and 54.8% regularly consume snacks and desserts, while only 57.4% consume fruits and just 27.1% consume vegetables. These imbalanced patterns persist into school age and contribute to the increasing burden of childhood obesity.

Excess weight gain during the first two years of life is a well-established predictor of obesity in later stages [8]. A range of early-life factors, including parental feeding practices, screen time, food availability, and dietary exposures, play a critical role in shaping children’s eating behaviors and self-regulation capacities [9,10,11]. These environmental influences interact with children’s emerging appetitive traits, potentially contributing to excessive energy intake and unhealthy dietary patterns [12,13]. It is important to explore behavioral characteristics, such as appetitive traits, that may predispose children to early feeding challenges. Understanding how these traits relate to actual dietary patterns can offer insight into the psychosocial domain of feeding, particularly during toddlerhood, when caregiver responsiveness, mealtime structure, and the emotional climate of feeding interactions are known to shape long-term eating trajectories [14]. Given the limited evidence from underrepresented populations such as Mexican toddlers, examining these behavioral–dietary relationships is essential for informing culturally relevant strategies that support healthy development from the start.

While environmental factors play a significant role in obesity risk, they do not act in isolation. The growing consensus around the Behavioral Susceptibility Theory (BST) suggests that childhood obesity emerges from a complex interplay between genetic predisposition and an “obesogenic” environment [15]. According to the BST, inherited differences in appetite serve as behavioral mediators of genetic susceptibility, influencing how individuals respond to their dietary environment. Variations in eating behaviors, or appetitive traits, can be identified as early as three months of age and may contribute to either excessive weight gain or insufficient weight gain during infancy. This perspective underscores the need for a multidimensional approach to obesity prevention and intervention, integrating both biological and environmental determinants while addressing broader food security and public health challenges [8,15].

Appetitive traits can be classified into two categories: food approach traits, which drive increased food intake (e.g., Food Responsiveness, Emotional Overeating, and Enjoyment of Food); and food avoidant traits, which are associated with reduced food intake (e.g., Satiety Responsiveness, Emotional Undereating, Food Fussiness, and Slowness in Eating). These traits can be assessed using psychometric questionnaires, which are cost-effective and easy to administer, providing valuable insights into how young children interact with food. The Child Eating Behavior Questionnaire-Toddler version (CEBQ-T) is the most comprehensive and validated psychometric tool for assessing eating behaviors in children aged one to three years [16,17].

Analyzing dietary patterns offers a comprehensive framework for understanding overall diet quality, as it captures the synergistic effects of nutrients, food groups, and eating behaviors that are often missed in traditional nutrient-based frameworks [18]. This approach reflects how individuals eat in real-life contexts and is particularly valuable when studying young children, whose eating habits are shaped by multiple interacting factors. Dietary patterns characterized by a high consumption of ultra-processed and fast foods have been associated with increased energy intake, higher body fat percentage, and elevated BMI z-scores in children, whereas patterns rich in fruits, vegetables, and minimally processed foods are consistently linked to healthier body composition and improved health outcomes [19,20,21].

Although these associations are well documented in older children, evidence on the impact of dietary patterns in infants and toddlers, particularly those under 24 months, remains limited and somewhat inconsistent [22]. However, emerging research suggests that early feeding practices and individual differences in appetite regulation may play a significant role in shaping these patterns. For example, parental strategies such as using food for emotional regulation or applying health-based restrictions have been positively associated with a higher consumption of ultra-processed foods (UPFs) in preschool-aged children, while monitoring behaviors appear to reduce intake [12]. These findings highlight the importance of considering both behavioral and dietary dimensions in early childhood nutrition research, especially when examining populations at risk for suboptimal dietary patterns and future obesity.

Additionally, prospective evidence from the Generation XXI birth cohort indicates that higher UPFs consumption at age four is associated with increased Food Responsiveness by age seven, an appetitive trait linked to heightened reactivity to food cues and greater obesity risk. Interestingly, higher UPFs intake was also associated with increased Food Fussiness and Satiety Responsiveness, which are food avoidant traits. These latter associations may reflect displacement of more satiating foods or changes in food reward processing rather than direct pathways to excess intake [23].

In line with these findings, certain appetitive traits have been identified as predictors of dietary patterns. For example, research indicates that children with higher Food Responsiveness at 16 months tend to eat more frequently by 21 months but do not necessarily consume larger meals. Conversely, children with lower Satiety Responsiveness tend to consume larger meal portions but do not eat as frequently. Additionally, among children aged four to 12 years, a preference for non-essential foods and poor dietary diversity has been associated with higher Food Fussiness, highlighting the potential influence of eating behaviors on dietary quality and health outcomes [24].

In Mexico, feeding difficulties have been observed as early as infancy, often intensifying during the second year of life and persisting into early childhood [25]. Early dietary intake is critical for metabolic programming and represents a sensitive window during which the establishment or correction of feeding behaviors and dietary patterns may influence lifelong health outcomes [26,27]. Given that eating behaviors formed in early childhood often persist over time, early identification and support for families experiencing feeding challenges are essential for promoting healthy trajectories and preventing chronic disease [28,29].

Therefore, the aim of this study was to investigate the relationship between appetitive traits and dietary patterns in children aged 12 to 36 months in Guadalajara, México. Given existing evidence on the role of appetitive traits in shaping eating behaviors, we hypothesize that a higher presence of food approach traits or a lower presence of food avoidant traits would be associated with less healthy dietary patterns, which are characterized by a higher intake of energy-dense, UPFs and a lower consumption of nutrient-dense dietary patterns.

## 2. Materials and Methods

### 2.1. Participants and Procedures

This analytical cross-sectional study employed a non-probabilistic, consecutive case sampling strategy. Participants were recruited from four sources: (1) the Hospital Civil de Guadalajara Dr. Juan I. Menchaca; (2) private pediatric practices in the Guadalajara metropolitan area; (3) the Trompo Mágico Museum, a site frequently visited by children in the target age range; and (4) social media networks of professionals specializing in maternal and child nutrition. Data collection methods varied by recruitment site: in hospitals and private practices, caregivers were approached in person and completed surveys either face-to-face or by telephone; at the Trompo Mágico Museum, participants were approached in person and interviewed via telephone; and those recruited through social media were contacted and surveyed entirely by telephone once they had consented to participate in the study. While face-to-face interviews are generally preferred, telephone interviews were used when in-person data collection was not feasible, particularly for participants recruited via social media and at the museum site. All interviewers followed a standardized protocol to ensure consistency across both modalities.

Participant recruitment was conducted between January and September 2022. All mothers or primary caregivers were interviewed either by A.S.G.-B. or by a final-year undergraduate nutrition student trained in standardized administration. Depending on the recruitment setting, the full questionnaire was administered either face-to-face or by telephone. Inclusion criteria for participation were (1) full-term birth; (2) birth weight appropriate for gestational age; (3) apparent good health status; and (4) maternal education level of at least completed primary school. The minimum required sample size (n = 137) was estimated using a Pearson correlation coefficient of *r* = 0.26 based on a previous study [24], with 80% power and a 5% significance level, which was calculated via UCSF’s sample size calculator [30]. To account for potential attrition, a 20% increase was applied. The final sample (n = 243) exceeded this target due to available time and resources and to enhance statistical robustness.

### 2.2. Sociodemographic Variables

Sociodemographic variables collected for this study included the toddler’s age (in months) and sex (feminine, masculine) as well as the caregiver’s age (in years). Caregivers also reported their highest level of education, which was categorized as basic education, high school/technical diploma, or university/postgraduate degree. Family type was recorded as either nuclear or other configurations such as mono-parental, extended and joint families. Marital status was categorized as married/cohabitation or divorced/separated/single. Socioeconomic variables included monthly household income and monthly expenses, both expressed in Mexican pesos, and reported using the median and interquartile range (MI [RI]) due to non-normal distribution.

### 2.3. Appetitive Traits

The children’s appetitive traits were measured the Child Eating Behavior Questionnaire—Toddler version (CEBQ-T) [17], which was validated for the Mexican population as the CEBQ-T-Mex [16]. This psychometric questionnaire consists of 26 items rated on a Likert scale ranging from 1 (“…”) to 5 (“…”), generating quantitative variables that correspond to the mean scores of each of the six constructs measured by the CEBQ-T.

The questionnaire measures three food approach traits: (1) Food Responsiveness (e.g., “My child is always asking for food”); (2) Emotional Overeating (e.g., “My child eats more when angry, impatient, or bored”); and (3) Enjoyment of Food (e.g., “My child loves food”). Additionally, it also measures three food avoidance traits: (4) Satiety Responsiveness (e.g., “My child becomes full before finishing a meal”), (5) Food Fussiness (e.g., “At first, my child rejects new foods”), and (6) Slowness in Eating (e.g., “It takes my child more than 30 min to finish a meal”). The internal consistency of the subscales was assessed using Cronbach’s alpha coefficients.

### 2.4. Dietary Patterns

To identify the dietary patterns of the participants, a qualitative food frequency questionnaire based on food groups was used [31]. This instrument included 33 food groups and was developed based on previous studies that assessed dietary patterns in toddlers [32,33]. Although the questionnaire was not formally validated in the target population, it was reviewed by a panel of nutritionists from the advisory committee (C.H.-A., C.C.-P., J.G.-T., and D.C.Á.-Z.) and piloted with 10 caregivers of toddlers aged 12 to 36 months. The pilot used a think-aloud methodology to assess face validity and ensure comprehensibility [34]. Minor adjustments were made based on participant feedback prior to full implementation.

Weekly consumption frequencies were calculated for each food group based on participants’ responses. These groups were then categorized to distinguish between healthier and less healthy options, following established criteria [33]. Dietary patterns were derived using PCA, which is a widely applied method in nutritional epidemiology that reduces complex dietary data into interpretable components by identifying correlations among food groups [35]. This data-driven approach provides insight into population-specific eating patterns and their potential links to health outcomes, offering valuable support for the design of targeted public health strategies and nutritional interventions.

### 2.5. Statistical Analysis

All statistical analyses were completed using SPSS version 25.0. No missing data were reported, as all questionnaires were administered through direct interviews, allowing for complete and consistent data collection.

The means and standard deviations for each subscale of the CEBQ-T were calculated by averaging the item scores corresponding to each trait. Skewness and kurtosis values were examined for all CEBQ-T-Mex subscales and indicated non-normal distributions. However, given the large sample size and in accordance with the Central Limit Theorem, parametric tests (Pearson correlations) were employed, as they are generally robust under such conditions and yield results comparable to those of non-parametric tests [36].

For additional qualitative analysis and to facilitate interpretation, each of the six appetitive trait subscales was transformed into a categorical variable using a cutoff score of 3, following a previously published method [37]. Scores greater than 3 on food approach subscales (e.g., Food Responsiveness, Emotional Overeating) were classified as “high”, while scores less than 3 on food avoidance subscales (e.g., Satiety Responsiveness, Slowness in Eating) were also classified as “high”. Scores equal to 3 were excluded from the categorical analysis to improve interpretability and potential clinical applicability.

PCA was used to identify dietary patterns based on food group consumption. Food groups with low variability (i.e., >70% of identical responses or “never” responses) were excluded, resulting in 24 food groups included in the final analysis. PCA was conducted using SPSS with orthogonal Varimax rotation to extract independent components [38]. The number of components retained was based on the scree plot and the rotated component matrix, using an eigenvalue threshold of ≥1.5 in line with established criteria for samples > 200 [38,39,40]. Food groups with factor loadings ≥ 0.30 were considered significant contributors [33].

The suitability of the data for PCA was confirmed using the Kaiser–Meyer–Olkin (KMO) measure and Bartlett’s test of sphericity [38]. Each participant received a factor score for each pattern, which was calculated as the sum of standardized weekly intake multiplied by the respective factor loading. To facilitate qualitative analysis, factor scores were divided into tertiles. Participants in the third tertile were considered to have the highest adherence to each dietary pattern, enabling both quantitative and categorical analyses of pattern adherence.

To examine the relationship between appetitive traits and dietary patterns, Pearson correlation analyses were conducted using continuous scores for both variables. To further explore these associations when both appetitive traits and dietary patterns were categorized, Chi-square tests were performed. Where significant associations were identified, post hoc Z-tests with Bonferroni correction were applied along with odds ratio analyses to estimate the strength of associations between specific traits and adherence to dietary patterns.

## 3. Results

### 3.1. Sociodemographic Variables

A total sample of 243 toddlers aged 12 to 36 months was included in the study. The mean age of the toddlers was 22.4 ± 7.1 months with a median of 22.0 months and an interquartile range (IQR) of 13 months. Of the total sample, 117 (48.1%) were female and 126 (51.9%) were male (Table 1).

When dividing the sample by age group, 143 participants (58.8%) were between 12 and 24 months old, and 100 participants (41.2%) were between 25 and 36 months. Among primary caregivers, a high proportion reported higher education (university or postgraduate level) (74.1%). The mean age of primary caregivers was 30.5 ± 5.2 years with a median of 30.0 years (IQR = 8).

Table 1 presents the sociodemographic characteristics of toddlers and their primary caregivers according to recruitment site. The majority of caregivers reported belonging to a nuclear family (79.8%) and being married or living with a partner (90.1%). The median monthly household income was 25,000 Mexican pesos (IQR = 26,000), and the median monthly household expenses were 6800 Mexican pesos (IQR = 5200).

Significant associations were found between the Trompo Mágico, social media, and private practice groups in relation to parental education level. A post hoc Z-test with Bonferroni correction revealed that the Trompo Mágico group differed significantly from those recruited via social media and private pediatric practices (*p* < 0.05). Specifically, participants from the Trompo Mágico site had a lower proportion of caregivers with higher education and a higher proportion with only basic education compared to the other two groups. Fisher’s exact test revealed significant associations between Trompo Mágico, social media, and private practice groups with respect to the marital status of the primary caregivers. A post hoc Z-test with Bonferroni correction indicated that the differences among groups were specifically related to the proportion of single caregivers. Private pediatric practices differed significantly from both the social media and Trompo Mágico groups (*p* < 0.05), as no single caregivers were reported in the private practice group.

### 3.2. Appetitive Traits

Table 2 shows that among the appetitive traits assessed, Enjoyment of Food had the highest mean and median scores, while Emotional Overeating had the lowest ($\overset{-}{X}$ = 4.16 ± 0.67 vs. $\overset{-}{X}$ = 1.72 ± 0.72). All appetitive trait subscales demonstrated acceptable internal consistency with Cronbach’s alpha values equal to or greater than 0.70 (Table 1).

When appetitive traits were transformed into categorical variables, the majority of participants exhibited low Emotional Overeating (94.4%) and high Enjoyment of Food (Table 3).

### 3.3. Dietary Patterns

PCA was conducted using the 24 food groups from the food frequency questionnaire. The KMO measure of sampling adequacy was 0.710, and Bartlett’s test of sphericity was significant (*p* < 0.001), indicating that the data met the assumptions required for this type of analysis.

The scree plot revealed an inflection point near an eigenvalue of 1.5, guiding the retention of three principal components. These components, interpreted as dietary patterns, were labeled: Processed; Healthy; and Dietary Transition/Modern Mexican. Collectively, they accounted for 32.5% of the total variance, which is considered acceptable in dietary pattern analysis [41] (Table 4).

The Processed pattern was characterized by a high intake of refined grains, processed meats, sauces and condiments, industrialized baby foods, sweetened dairy beverages, sugar-sweetened drinks, and natural juice. In contrast, the Healthy pattern included a higher consumption of whole grains and tubers, vegetables, fruits, legumes, fish, milk, plain yogurt, eggs, seeds, and unsweetened beverages. The third pattern, Dietary Transition/Modern Mexican, combined traditional and processed elements, including corn-based products, red and white meats, sweet or savory cereals, snacks, and desserts (Table 4).

When pattern scores were categorized into tertiles, the largest proportion of participants (61.7%) was classified as adherent to the Healthy pattern, which was followed by 24.7% adhering to the Processed pattern and 13.6% to the Dietary Transition/Modern Mexican pattern.

### 3.4. Appetitive Traits and Dietary Patterns

Table 5 presents the associations between appetitive traits and dietary patterns. A direct and significant relationship was identified between the food approach traits Food Responsiveness and Emotional Overeating with the Processed dietary pattern (*p* < 0.001), while they were negatively and significantly associated with Enjoyment of Food (*p* = 0.010).

Enjoyment of Food was directly and significantly associated with the Healthy dietary pattern (*p* < 0.001), whereas Food Fussiness (*p* < 0.001) and Satiety Responsiveness (*p* = 0.011) were inversely and significantly associated with this pattern (Table 5).

The Dietary Transition/Modern Mexican pattern was significantly correlated with all food approach traits. Notably, it showed a negative correlation with Enjoyment of Food (*p* = 0.020), suggesting that lower enjoyment may be linked to adherence to this pattern. Additionally, significant and positive correlations were observed between this pattern and the food avoidance traits Food Fussiness (*p* = 0.001) and Satiety Responsiveness (*p* = 0.047) (Table 5).

Table 6 presents the distribution of each identified dietary pattern according to levels of appetitive traits. Using the Chi-square test, a significant association was found between Food Fussiness and dietary patterns (*p* < 0.001). Post hoc Z-tests with Bonferroni correction revealed that the Dietary Transition/Modern Mexican pattern was significantly more prevalent among children with higher Food Fussiness compared to those adhering to the Processed or Healthy patterns.

Based on these results, a series of odds ratio analyses was conducted to further examine the likelihood of dietary pattern adherence by appetitive trait category. Toddlers with high Food Fussiness were significantly less likely to follow a Healthy dietary pattern [OR = 0.4; 95% CI: 0.2–0.8; *p* = 0.003]. In contrast, toddlers with high Food Responsiveness were found to have 2.2 times greater odds of adhering to a Processed dietary pattern [OR = 2.2; 95% CI: 1.1–4.5; *p* = 0.026]. Additionally, those with high Food Fussiness had a 5.2 times higher risk of following the Dietary Transition/Modern Mexican pattern [OR = 5.2; 95% CI: 2.4–11.6; *p* < 0.001] (Supplementary Materials Table S1).

## 4. Discussion

This study aimed to explore the relationship between appetitive traits and dietary patterns in a sample of Mexican toddlers aged 12 to 36 months. Using data collected through caregiver-reported questionnaires and food frequency assessments, we identified three distinct dietary patterns: Processed, Healthy, and Dietary Transition/Modern Mexican, which were derived through PCA. We found that most appetitive traits, particularly Food Fussiness, Food Responsiveness, and Satiety Responsiveness, were significantly associated with these dietary patterns. Children with higher scores in food approach traits were more likely to adhere to energy-dense patterns, while food avoidance traits such as Food Fussiness were associated with a lower likelihood of following healthier diets. Notably, Slowness in Eating was the only trait not significantly associated with any dietary pattern.

To our knowledge, this is the first study in Mexico and among the first internationally to examine the relationship between appetitive traits and empirically derived dietary patterns in children under three years of age. These findings provide valuable insight into the behavioral underpinnings of early dietary exposures during a critical window for nutritional and developmental programming.

Among the appetitive traits assessed, Enjoyment of Food had the highest mean score, while Emotional Overeating had the lowest. This pattern aligns with prior findings in Mexican toddlers [16,42]. The low average score for Emotional Overeating may reflect developmental factors, as emotional regulation through eating typically consolidates at later ages [43], or it may relate to caregivers’ limited awareness of young children’s emotional triggers during meals [11]. Socioeconomic factors may also play a role: studies have shown that lower socioeconomic status is associated with higher Emotional Overeating scores [10], while our sample, largely composed of caregivers with higher education and income levels, may be less exposed to stress-related feeding environments.

The high prevalence of Enjoyment of Food could suggest a generally positive feeding environment within this sample. This is consistent with research showing that higher maternal education and responsive feeding practices are linked to greater Enjoyment of Food and reduced fussy eating [44]. Furthermore, the internal consistency of all appetitive trait subscales (Cronbach’s alpha ≥ 0.70) supports the reliability of caregiver reports [38]. These findings provide useful insight into the behavioral feeding profiles of toddlers in this context and highlight the potential value of using trait-based approaches to identify feeding styles that may support or hinder healthy dietary development [45].

Three distinct dietary patterns were identified in this sample of Mexican toddlers: a Processed pattern, characterized by a high intake of ultra-processed and sugar-sweetened foods; a Healthy pattern, rich in minimally processed foods such as fruits, vegetables, legumes, fish, and whole grains; and a Dietary Transition/Modern Mexican pattern, which included both traditional foods (e.g., corn-based products, red meats) and processed items (e.g., sweetened cereals and snacks). Together, these patterns explained 32.5% of the total dietary variance, which is a proportion considered acceptable in early childhood nutrition research [41].

The Processed pattern closely resembles the “Westernized” dietary patterns described in other studies of young children in Mexico and Australia [25,33], which is marked by the presence of sugary beverages, fast food, and industrialized baby snacks. This is concerning given the growing evidence linking UPFs consumption in early life to increased risk of overweight, cardiometabolic disease, and poor dietary diversity [46,47,48]. UPFs consumption has also been associated with increased obesity risk [49]. In contrast, the Healthy pattern aligns with global recommendations for young children’s diets [50,51,52] and mirrors patterns identified in preschoolers from other countries, which have been referred to variously as “prudent” [53], “health-conscious” [33], or “healthy” [54]. The Dietary Transition/Modern Mexican pattern reflects broader shifts in the Mexican food system, where traditional dietary staples coexist with an increasing availability of processed and energy-dense foods [55,56].

### 4.1. Processed Dietary Pattern and Appetitive Traits

The associations observed between appetitive traits and dietary patterns in this study offer important insight into early behavioral susceptibility to obesogenic environments. Toddlers with higher Food Responsiveness and Emotional Overeating scores were significantly more likely to adhere to a Processed dietary pattern, which is rich in UPFs. This finding aligns with the Behavioral Susceptibility Theory, which posits that children with heightened reactivity to food cues are more vulnerable to the effects of food environments that promote energy-dense, palatable food consumption [8,15]. Although most existing evidence comes from studies in older children and adolescents, similar associations have been reported. For instance, UPFs consumption has been linked to higher adiposity and unfavorable cardiometabolic markers in school-aged children and teens [13,20,21]. Vedovato et al. (2021) further showed that higher UPFs intake in early childhood is prospectively associated with increased Food Responsiveness, while Prates et al. (2022) identified parenting practices that contribute to UPFs consumption in preschoolers [12,13].

Our findings extend this body of research by identifying these associations in toddlers aged 12 to 36 months, which is an underrepresented age group in behavioral nutrition studies. In addition, longitudinal evidence from Rauber et al. (2015) shows that early UPFs consumption is linked to adverse lipid profiles later in childhood [57]. Syrad et al. (2016) also demonstrated that children with higher Food Responsiveness tend to eat more frequently, which may increase their exposure to UPFs when these foods are used as convenient or accepted snacks [58]. Taken together, these findings suggest that behavioral predispositions such as heightened food responsiveness not only affect intake frequency but may also shape a preference for processed dietary patterns, reinforcing less healthy food choices from a young age.

### 4.2. Healthy Dietary Pattern and Appetitive Traits

In this study, the Healthy dietary pattern—characterized by a higher intake of vegetables, fruits, legumes, whole grains, fish, and unsweetened beverages—was positively associated with Enjoyment of Food and inversely associated with Food Fussiness and Satiety Responsiveness. These findings are broadly consistent with previous literature linking food approach and avoidance traits to food variety and diet quality. For example, Jani et al. (2020) reported that higher Food Fussiness scores were associated with lower fruit and vegetable consumption and a greater intake of non-core foods in Australian children aged 4 to 12 years [24]. Similarly, Cole et al. (2017) identified an inverse relationship between food neophobia or fussiness and vegetable intake in children under 30 months [59]. Although most existing studies have focused on older children, our results extend these associations to toddlers in a different cultural context.

Higher Enjoyment of Food was associated with greater adherence to the Healthy pattern, suggesting that positive affective responses to eating may promote an acceptance of nutrient-dense foods early in life. While direct evidence linking Enjoyment of Food to healthy dietary patterns remains limited, it may reflect broader influences from the feeding environment. Caregivers in our sample generally had higher education and income levels, which are factors associated with a greater use of responsive feeding practices [60]. Responsive feeding, characterized by attention to hunger and satiety cues and avoidance of coercive strategies, has been associated with increased Enjoyment of Food and healthier eating behaviors [44,61].

Although the positive association between Enjoyment of Food and healthy patterns has not been extensively studied in toddlers, our results are consistent with emerging evidence linking early appetitive traits to diet quality trajectories. For instance, research from older populations has shown that lower levels of food enjoyment are associated with lower dietary variety and higher UPFs [12,13]. However, much of this research has been conducted in school-aged children or adolescents [19,20,21], highlighting the need for further studies focused on younger age groups to better understand these early pathways.

### 4.3. Dietary Transition/Modern Mexican Dietary Pattern and Appetitive Traits

The Dietary Transition/Modern Mexican pattern, combining traditional staples with processed foods, was positively associated with Food Responsiveness and Emotional Overeating. These traits have been consistently linked to higher adiposity in young children [62], and similar snack-rich dietary patterns have been associated with increased obesity risk in toddlers [54]. Interestingly, this pattern was inversely associated with Enjoyment of Food, contrasting with findings from the Netherlands where higher enjoyment was linked to greater snack intake in toddlers [63]. In our context, lower Enjoyment of Food may lead caregivers to offer more palatable, processed foods to encourage intake, which is a strategy observed in qualitative studies among Mexican–American families [64].

Positive associations were also found between the Dietary Transition/Modern Mexican pattern and Satiety Responsiveness and Food Fussiness. Caregivers may accommodate avoidant or selective eating behaviors by relying on more accepted, often processed foods, rather than persisting with repeated exposures to healthier options (Smith et al., 2017) [65]. Notably, toddlers with high Food Fussiness had a fivefold greater likelihood of adhering to this dietary pattern. These findings align with longitudinal evidence showing that early fussiness and Emotional Overeating predict lower diet quality and higher sugary beverage intake in young adulthood [66].

Chi-square and odds ratio analyses further reinforced the role of Food Fussiness as a significant behavioral barrier to healthy eating in early childhood. Toddlers with higher Food Fussiness scores were significantly less likely to adhere to a Healthy dietary pattern and had 5.2 times greater odds of following the Dietary Transition/Modern Mexican pattern. These findings align with previous studies showing that Food Fussiness is negatively associated with fruit and vegetable intake and overall diet quality in children [24,59]. Although most previous research has been conducted in older children and adolescents [19,20,21], our findings suggest that the influence of fussy eating on dietary pattern adherence emerges as early as the toddler years. Early identification and intervention strategies targeting fussiness may be critical for promoting healthier dietary trajectories in at-risk populations.

### 4.4. Strengths and Limitations

This study is one of the first to examine the relationship between appetitive traits and dietary patterns in Mexican toddlers, which is an underrepresented population in behavioral nutrition research. The use of the CEBQ-T, an important measuring tool for assessing appetitive traits in young children that has been validated in Mexico, adds value to the reliability and comparability of the findings across settings. In addition, the application of PCA to identify dietary patterns allowed for a holistic understanding of habitual food group combinations, which may be more informative than nutrient-level analyses for early behavioral research.

Nevertheless, several limitations should be considered when interpreting these findings. First, the cross-sectional design limits the ability to infer causality between appetitive traits and dietary patterns. While associations were observed, appetitive traits are shaped by complex, multifactorial influences, including genetic, behavioral, and environmental factors that interact over time to impact eating behaviors [15,58]. Second, the study relied on caregiver self-report measures, which may be subject to recall or reporting bias. While such limitations are common in retrospective designs, future research could benefit from prospective methodologies or the inclusion of direct mealtime observations to improve accuracy and reduce bias in assessing children’s eating behaviors and dietary intake [58].

This study was conducted in an urban region of western Mexico, and findings may not be generalizable to all cultural or socioeconomic settings. However, given that appetitive traits have demonstrated cross-cultural relevance [24,25,42], these results may provide a useful basis for future comparative studies in other populations.

Additionally, participants were recruited from three distinct settings, a public hospital, private pediatric practices, and social media, which differed in their sociodemographic characteristics. While this allowed for a diverse sample, it also introduces potential heterogeneity that may have influenced observed associations. Moreover, participants were not randomly selected from a defined population but were instead volunteers, which may have introduced selection bias, particularly in the private practice group, where caregivers may have had greater health literacy and nutritional awareness [67]. As a result, the generalizability of the findings is limited.

Dietary intake was assessed using a qualitative food group frequency questionnaire. While this method does not allow for precise nutrient estimations, it is a validated tool for identifying habitual food group combinations in young children [33,54]. However, we acknowledge that other aspects of early eating behavior, such as meal frequency, duration, and the psychosocial feeding context, may also influence both dietary patterns and developmental outcomes and thus should be considered in future studies [11,58,68].

In terms of statistical methodology, some decisions in the PCA, such as the selection of food groups, the choice of Varimax rotation, and the naming of dietary patterns, involve researcher judgment, which is a common and acknowledged limitation in pattern analysis. Despite this, efforts were made to ground these decisions in both statistical criteria and culturally relevant dietary knowledge.

Recent work by Kininmonth et al. (2023) highlights the importance of considering emotional functioning and caregiver–child feeding dynamics when interpreting early eating behaviors [11]. Their findings suggest that child irritability, emotional reactivity, and caregiver emotional availability may mediate or moderate the relationship between appetitive traits and later health outcomes. These psychosocial processes may help explain our finding that toddlers with higher Food Fussiness were more likely to consume UPFs and adhere to non-nutritive dietary patterns, potentially reflecting coping strategies by caregivers facing mealtime conflict or food refusal.

If unaddressed, these behaviors may evolve into feeding difficulties of clinical concern, affecting nutrient adequacy, growth, and caregiver well-being. The psychosocial domain of the Pediatric Feeding Disorder (PFD) framework [14] highlights how maladaptive caregiver–child interactions and environmental factors can influence feeding outcomes. In the presence of fussiness, caregivers may prioritize food acceptance over quality, adopting more permissive feeding practices that reinforce the consumption of energy-dense, nutrient-poor foods [61,69,70]. This pattern is consistent with evidence linking unstructured or emotionally charged feeding practices to risks of both undernutrition and overnutrition in early childhood [14,71].

To better understand the clinical relevance of these associations, future longitudinal studies are warranted to track developmental trajectories, identify modifiable risk factors, and examine how appetitive traits and dietary patterns interact with growth indicators such as BMI z-scores and other anthropometric data. Such work could inform culturally and contextually tailored interventions to support early childhood nutrition and health [37,45].

## 5. Conclusions

The findings of this study contribute to a better understanding of the relationship between appetitive traits and dietary patterns in children aged 12 to 36 months. In this sample, higher Enjoyment of Food was most strongly associated with adherence to a Healthy dietary pattern, whereas elevated scores in Food Fussiness and Satiety Responsiveness were linked to a lower likelihood of following such a pattern. Moreover, toddlers who scored higher in Food Responsiveness, Emotional Overeating, Satiety Responsiveness, and Food Fussiness were more likely to adhere to Processed or Dietary Transition/Modern Mexican patterns, which were both characterized by a greater consumption of ultra-processed and energy-dense foods.

These results suggest that identifying the appetitive profile of children during the toddler years may be a valuable tool in promoting responsive feeding practices, which involve recognizing and appropriately responding to hunger and satiety cues. Such practices have been associated with the development of healthier eating behaviors and may reduce the risk of feeding difficulties and obesity later in life. Appetitive traits observed, particularly those involving food avoidance traits and a preference for less nutritious diets, may reflect underlying psychosocial feeding challenges that warrant further attention.

These findings open the door for future research to explore tailored interventions based on children’s appetitive traits. By aligning feeding strategies with a child’s behavioral tendencies, such interventions may be more effective in guiding families toward the establishment of healthy and sustainable dietary patterns from early childhood.

## Figures and Tables

**Table 1:** Descriptive characteristics of toddlers and primary caregivers (n = 243).

	Participant Recruitment Site				
	Hospital Civil de Guadalajara Dr. Juan I. Menchaca	Private Pediatric Practices	Maternal & Child Nutrition Professionals’ Social Media	Trompo Mágico Museum	Total
	M (SD) or n (%)	M (SD) or n (%)	M (SD) or n (%)	M (SD) or n (%)	M (SD) or n (%)
Toddlers					
Age (months)	22.0 (5.9)	22.1 (6.9)	20.3 (7.3)	24.9 (6.9)	22.4 (7.1)
Sex					
Feminine	10 (50.0)	35 (42.7)	35 (51.5)	37 (50.7)	117 (48.1)
Masculine	10 (50.0)	47 (57.3)	33 (48.5)	36 (49.3)	126 (51.9)
Principal caregiver					
Age (years)	26.1 (4.3)	30.4 (4.4)	31.1 (4.9)	31.2 (5.8)	30.5 (5.2)
Education					
Basic education	8 (40.0)	2 (2.4)	5 (7.4)	9 (12.3)	24 (9.9)
High school/Technical diploma	8 (40.0)	10 (12.2)	4 (5.9)	17 (23.3)	39 (16.0)
University/Postgraduate degree	4 (20.0)	70 (85.4)	59 (86.7)	47 (64.4)	180 (74.1)
Family type					
Nuclear	11 (55)	69 (84.1)	54 (79.4)	60 (82.2)	194 (79.8)
Other	9 (45)	13 (15.9)	14 (20.6)	13 (17.8)	49 (20.2)
Marital status					
Married/Cohabitation	15 (75)	81 (50.0)	58 (85.2)	65 (89.0)	219 (90.1)
Divorced/Separated/Single	5 (25)	1 (1.2)	10 (13.2)	8 (11.0)	24 (9.9)
Income *	MI (RI)	MI (RI)	MI (RI)	MI (RI)	MI (RI)
Monthly income	10,000 (16,000)	27,000 (145,000)	28,500 (141,000)	20,000 (146,000)	25,000 (26,000)
Monthly expenses	4000 (14,600)	6900 (13,500)	7950 (17,600)	6000 (40,800)	6800 (5200)

M (SD): mean (standard deviation); MI (RI): median and interquartile range; n (%): frequency (percentage); * Mexican pesos.

**Table 2:** Measures of central tendency and dispersion for CEBQ-T constructs in toddlers (n = 243).

Appetitive Trait Subscales	Mean	SD	Median	IQR	Alfa Cronbach
Food approach traits					
Food Responsiveness	2.43	0.82	2.00	1.50	0.745
Emotional Overeating	1.72	0.72	1.00	1.00	0.78
Enjoyment of Food	4.16	0.67	4.50	1.00	0.82
Food avoidance traits					
Satiety Responsiveness	2.67	0.63	3.00	1.00	0.70
Food Fussiness	2.34	0.86	2.00	1.50	0.88
Slowness in Eating	2.58	0.74	2.50	1.00	0.70

SD: standard deviation; IQR: interquartile range.

**Table 3:** Categorical distribution of appetitive traits by recruitment site of toddlers (n = 243).

Appetitive Trait Subscales		Recruitment Site					
		Hospital Civil	Private Practices	Social Media	Trompo Mágico Museum	Total	*p* *
		n (%)	n (%)	n (%)	n (%)	n (%)	
Food approach traits							
Food Responsiveness	High	3 (6.8)	11 (25.0)	12 (27.3)	18 (40.9)	44 (100)	0.179
	Low	12 (7.0)	64 (37.2)	48 (27.9)	48 (27.9)	172 (100)	
Emotional Overeating	High	2 (15.4)	5 (38.5)	3 (23.1)	2 (23.1)	13 (100)	0.867
	Low	17 (7.7)	76 (34.5)	63 (28.6)	64 (29.1)	220 (100)	
Enjoyment of Food	High	18 (8.1)	75 (33.9)	62 (28.1)	66 (29.9)	221 (100)	0.580
	Low	1 (11.1)	2 (22.2)	2 (22.2)	4 (44.4)	9 (100)	
Food avoidance traits							
Satiety Responsiveness	High	4 (6.9)	24 (41.4)	15 (25.9)	15 (25.9)	58 (100)	0.287
	Low	13 (8.0)	49 (30.1)	47 (28.8)	54 (33.1)	163 (100)	
Food Fussiness	High	3 (6.3)	20 (41.7)	9 (18.8)	16 (33.3)	48 (100)	0.200
	Low	15 (8.2)	57 (31.0)	56 (30.4)	56 (30.4)	184 (100)	
Slowness in Eating	High	5 (8.5)	21 (35.6)	18 (30.5)	15 (25.4)	59 (100)	0.701
	Low	12 (7.5)	53 (33.3)	44 (27.7)	50 (31.4)	159 (100)	

* *p*-values correspond to Chi-square tests comparing private practices, social media, and Trompo Mágico groups. The Hospital Civil group was excluded from this analysis due to its small sample size.

**Table 4:** Factor loadings for each food group across the three identified dietary patterns.

No.	Food Group	Processed	Healthy	Dietary Transition/ Modern Mexican
1	Refined grains	0.334	—	—
2	Whole grains and tubers	—	0.404	—
3	Corn products	—	—	0.347
4	Vegetables	—	0.554	—
5	Fresh fruits	—	0.655	—
6	Legumes	—	0.331	—
7	Beef or pork	—	—	0.692
8	Chicken or turkey	—	—	0.362
9	Fish and seafood	—	0.429	—
10	Eggs	—	0.494	—
11	Processed meats	0.698	—	—
12	Cow’s milk and plain yogurt	—	0.418	—
13	Sweetened dairy drinks	0.625	—	—
14	Cheese	0.363	0.461	—
15	Fats	—	0.384	—
16	Seeds and derived products	—	0.519	—
17	Sauces and condiments	0.666	—	—
18	Industrialized baby foods	0.314	—	—
19	Sweet or savory breakfast cereals (processed)	0.432	—	0.607
20	Snacks, sweets, and desserts	0.424	—	0.632
21	Fast food	0.627	—	—
22	Sugar-sweetened beverages	0.690	—	—
23	Unsweetened beverages	—	0.307	—
24	Natural juice	0.405	—	—
	Explained variance (%)	15.447%	10.181%	6.892%

**Table 5:** Pearson correlations between mean appetitive trait scores and dietary pattern cores in toddlers (n = 243).

Appetitive Trait Subscales	Processed Pattern		Healthy Pattern		Dietary Transition/Modern Mexican Pattern	
	r	*p*-Value	r	*p*-Value	r	*p*-Value
Food approach traits						
Food Responsiveness	0.254	<0.001	0.115	0.075	0.144	0.025
Emotional Overeating	0.224	<0.001	0.054	0.401	0.138	0.031
Enjoyment of Food	−0.165 *	0.010	0.269	<0.001	−0.149	0.020
Food avoidance traits						
Satiety Responsiveness	−0.052	0.417	−0.162	0.011	0.127	0.047
Food Fussiness	0.084	0.191	−0.241	0.000	0.210	0.001
Slowness in Eating	−0.079	0.219	−0.074	0.252	0.114	0.076

r = Pearson correlation coefficient. * For Enjoyment of Food and the Processed pattern, the Spearman correlation coefficient was used due to non-normal distribution.

**Table 6:** Association between categorized appetitive traits levels and dietary patterns in toddlers (n = 243).

Appetitive Trait Subscales		Dietary Pattern				
		Processed	Healthy	Transition/ Modern Mexican	Total	*p*-Value *
		n (%)	n (%)	n (%)	n (%)	
Food Approach Traits						
Food Responsiveness	High	16 (36.4)	24 (54.5)	4 (9.1)	44 (100)	0.071
	Low	35 (20.3)	111 (64.5)	26 (15.1)	172 (100)	
Emotional Overeating	High	3 (23.1)	8 (61.5)	2 (15.4)	13 (100)	0.991
	Low	52 (23.6)	137 (62.3)	31 (14.1)	220 (100)	
Enjoyment of Food	High	52 (23.5)	141 (63.8)	28 (12.7)	221 (100)	0.196
	Low	2 (22.2)	4 (44.4)	3 (33.3)	9 (100)	
Food Avoidance Traits						
Satiety Responsiveness	High	13 (22.4)	34 (58.6)	11 (19.0)	58 (100)	0.378
	Low	40 (24.5)	104 (63.8)	19 (11.7)	163 (100)	
Food Fussiness ^a^	High	11 (22.9)	21 (43.8)	16 (33.3)	48 (100)	<0.001
	Low	44 (23.9)	124 (67.4)	16 (8.7)	184 (100)	
Slowness in Eating	High	13 (22.0)	35 (59.3)	11 (18.6)	59 (100)	0.273
	Low	43 (27.0)	99 (62.3)	17 (10.7)	159 (100)	

* *p*-value derived from Chi-square test. ^a^ Post hoc Z-test with Bonferroni correction: this proportion differs significantly (*p* < 0.05) from those in the Processed and Healthy patterns.

## Data Availability

Data are available from A.S.G.-B. upon reasonable request.

## Supplementary Materials

The following supporting information can be downloaded at https://www.mdpi.com/article/10.3390/nu17111814/s1, Table S1: Odds ratio analyses of dietary pattern adherence by appetitive trait category.

## Abbreviations

The following abbreviations are used in the manuscript:

BST	Behavioral Susceptibility Theory
KMO	Kaiser–Meyer–Olkin
PCA	principal component analysis
PFD	Pediatric Feeding Disorder
UPFs	ultra-processed foods

## References

[1] Lott M., Callahan E., Welker Duffy E., Story M., Daniels S. Healthy Beverage Consumption in Early Childhood: Recommendation from Key National Health and Nutrition Organizations. Consensus Statement. https://healthyeatingresearch.org/wp-content/uploads/2019/09/HER-HealthyBeverage-ConsensusStatement.pdf.

[2] Siega-Riz A.M., Deming D.M., Reidy K.C., Fox M.K., Condon E., Briefel R.R. (2010). Food Consumption Patterns of Infants and Toddlers: Where Are We Now?. J. Am. Diet. Assoc..

[3] USDA & USDHHS Dietary Guidelines for Americans 2025. Make Every Bite Count with the Dietary Guidelines. https://www.dietaryguidelines.gov/sites/default/files/2020-12/Dietary_Guidelines_for_Americans_2020-2025.pdf.

[4] Rebello-Britto P. Early Moments Matter for Every Child|UNICEF. https://www.unicef.org/sites/default/files/press-releases/glo-media-UNICEF_Early_Moments_Matter_for_Every_Child_report.pdf.

[5] American Academy of Pediatrics Toddler Food and Feeding. https://www.aap.org/en/patient-care/healthy-active-living-for-families/toddler-food-and-feeding/?srsltid=AfmBOoq7s5UAOamdKtz-z6dQGpfw4Ywyg7jxsCw14afT9-j_AZSek4fJ.

[6] CDC Tips for Mealtime Routines|Infant and Toddler Nutrition|CDC. https://www.cdc.gov/infant-toddler-nutrition/mealtime/index.html?utm_source=chatgpt.com.

[7] Shamah-Levy T., Lazcano-Ponce E.C., Cuevas-Nasu L., Romero-Martínez M., Gaona-Pineda E.B., Gómez-Acosta L.M., Mendoza-Alvarado L.R., Méndez-Gómez-Humarán I. Encuesta Nacional de Salud y Nutrición Continua 2023. Resultados Nacionales. https://insp.mx/resources/images/stories/2025/docs/250108_Ensanut_23.pdf.

[8] Llewellyn C.H., Kininmonth A.R., Herle M., Nas Z., Smith A.D., Carnell S., Fildes A. (2023). Behavioural Susceptibility Theory: The Role of Appetite in Genetic Susceptibility to Obesity in Early Life. Philos. Trans. R. Soc. B.

[9] Haines J., Haycraft E., Lytle L., Nicklaus S., Kok F.J., Merdji M., Fisberg M., Moreno L.A., Goulet O., Hughes S.O. (2019). Nurturing Children’s Healthy Eating: Position Statement. Appetite.

[10] Kininmonth A.R., Smith A.D., Llewellyn C.H., Fildes A. (2020). Socioeconomic Status and Changes in Appetite from Toddlerhood to Early Childhood. Appetite.

[11] Kininmonth A.R., Herle M., Haycraft E., Farrow C., Tommerup K., Croker H., Pickard A., Edwards K., Blissett J., Llewellyn C. (2023). Reciprocal Associations between Parental Feeding Practices and Child Eating Behaviours from Toddlerhood to Early Childhood: Bivariate Latent Change Analysis in the Gemini Cohort. J. Child. Psychol. Psychiatry.

[12] Prates C.B., Passos M.A.Z., Masquio D.C.L. (2022). Parental Feeding Practices and Ultra-Processed Food Consumption in Preschool Children. Rev. Nutr..

[13] Milhassi Vedovato G., Vilela S., Severo M., Rodrigues S., Lopes C., Oliveira A. (2021). Ultra-Processed Food Consumption, Appetitive Traits and BMI in Children: A Prospective Study. Br. J. Nutr..

[14] Goday P.S., Huh S.Y., Silverman A., Lukens C.T., Dodrill P., Cohen S.S., Delaney A.L., Feuling M.B., Noel R.J., Gisel E. (2019). Pediatric Feeding Disorder: Consensus Definition and Conceptual Framework. J. Pediatr. Gastroenterol. Nutr..

[15] Llewellyn C.H., Fildes A. (2017). Behavioural Susceptibility Theory: Professor Jane Wardle and the Role of Appetite in Genetic Risk of Obesity. Curr. Obes. Rep..

[16] Hunot-Alexander C., González-Toribio J., Vásquez-Garibay E.M., Larrosa-Haro A., Casillas-Toral E., Curiel-Curiel C.P. (2021). Validity and Reliability of the Baby and Child Eating Behavior Questionnaire, Toddler Version (Bebq-Mex and Cebq-t-Mex) in a Low Sociodemographic Sample Recruited in a Mexican Hospital. Behav. Sci..

[17] Herle M.P. The Aetiology of Emotional Eating in Childhood. https://discovery.ucl.ac.uk/id/eprint/10025834/.

[18] Lazarou C., Newby P.K. (2011). Use of Dietary Indexes among Children in Developed Countries. Adv. Nutr..

[19] Latorre-Millán M., Rupérez A.I., González-Gil E.M., Santaliestra-Pasías A., Vázquez-Cobela R., Gil-Campos M., Aguilera C.M., Gil Á., Moreno L.A., Leis R. (2020). Dietary Patterns and Their Association with Body Composition and Cardiometabolic Markers in Children and Adolescents: Genobox Cohort. Nutrients.

[20] Chen Z.H., Mousavi S., Mandhane P.J., Simons E., Turvey S.E., Moraes T.J., Subbarao P., Miliku K. (2025). Ultraprocessed Food Consumption and Obesity Development in Canadian Children. JAMA Netw. Open.

[21] Mescoloto S.B., Pongiluppi G., Domene S.M.Á. (2024). Ultra-Processed Food Consumption and Children and Adolescents’ Health. J. Pediatr..

[22] Stanford F.C., Taylor C., Hoelscher D.M., Anderson C.A.M., Booth S., Deierlein A., Fung T., Gardner C., Giovannucci E., Raynor H. (2024). Dietary Patterns with Ultra-Processed Food Dietary Patterns and Growth, Body Composition, and Risk of Obesity: A Systematic Review.

[23] da Costa M.P., Severo M., Oliveira A., Lopes C., Hetherington M., Vilela S. (2022). Longitudinal Bidirectional Relationship between Children’s Appetite and Diet Quality: A Prospective Cohort Study. Appetite.

[24] Jani R., Agarwal C.K., Golley P., Shanyar N., Mallan K., Chipchase L. (2020). Associations between Appetitive Traits, Dietary Patterns and Weight Status of Children Attending the School Kids Intervention Program. Nutr. Health.

[25] Deming D.M., Afeiche M.C., Reidy K.C., Eldridge A.L., Villalpando-Carrión S. (2015). Early Feeding Patterns among Mexican Babies: Findings from the 2012 National Health and Nutrition Survey and Implications for Health and Obesity Prevention. BMC Nutr..

[26] Koletzko B., Brands B., Grote V., Kirchberg F.F., Prell C., Rzehak P., Uhl O., Weber M. (2017). Long-Term Health Impact of Early Nutrition: The Power of Programming. Ann. Nutr. Metab..

[27] Moreno Villares J.M. (2016). Los Mil Primeros Días de Vida y La Prevención de La Enfermedad En El Adulto. Nutr. Hosp..

[28] Kochanska G., Woodard J., Kim S., Koenig J.L., Yoon J.E., Barry R.A. (2010). Positive Socialization Mechanisms in Secure and Insecure Parent–Child Dyads: Two Longitudinal Studies. J. Child Psychol. Psychiatry.

[29] Feinstein L., Sabates R., Sorhaindo A., Rogers I., Herrick D., Northstone K., Emmett P. (2008). Dietary Patterns Related to Attainment in School: The Importance of Early Eating Patterns. J. Epidemiol. Community Health.

[30] Kohn M.S.J. Sample Size Calculators for Designing Clinical Research. https://sample-size.net/.

[31] Pérez Rodrigo C., Aranceta J., Salvador G., Varela-Moreiras G., Pérez Rodrigo Fundación FIDEC C.C. (2015). Métodos de Frecuencia de Consumo Alimentario. Rev. Esp. Nutr. Comunitaria.

[32] Bell L.K., Golley R.K., Mauch C.E., Mathew S.M., Magarey A.M. (2019). Validation Testing of a Short Food-Group-Based Questionnaire to Assess Dietary Risk in Preschoolers Aged 3–5 Years. Nutr. Diet..

[33] Shi Z., Makrides M., Zhou S.J. (2018). Dietary Patterns and Obesity in Preschool Children in Australia: A Cross-Sectional Study. Asia Paxific J. Clin. Nutr..

[34] Banna J.C., Vera Becerra L.E., Kaiser L.L., Townsend M.S. (2010). Using Qualitative Methods to Improve Questionnaires for Spanish Speakers: Assessing Face Validity of a Food Behavior Checklist. J. Am. Diet. Assoc..

[35] Zhao J., Li Z., Gao Q., Zhao H., Chen S., Huang L., Wang W., Wang T. (2021). A Review of Statistical Methods for Dietary Pattern Analysis. Nutr. J..

[36] Wayne W.D. (2012). Bioestadística: Base Para El Análisis de Las Ciencias de La Salud.

[37] Hunot-Alexander C., Croker H., Fildes A., Johnson F., Beeken R.J. (2022). Brief “Appetitive Trait Tailored Intervention”: Development in a Sample of Adults with Overweight and Obesity. Behav. Change.

[38] Field A. (2024). Discovering Statistics Using IBM SPSS Statistics.

[39] Flores M.E., Rivera-Pasquel M., Macías N., Sánchez-Zamorano L.M., Rodríguez-Ramírez S., Contreras-Manzano A., Denova-Gutiérrez E. (2021). Dietary Patterns in Mexican Preschool Children Are Associated with Stunting and Overweight. Rev. Saude Publica.

[40] de Jonge E.A.L., Rivadeneira F., Erler N.S., Hofman A., Uitterlinden A.G., Franco O.H., Kiefte-de Jong J.C. (2018). Dietary Patterns in an Elderly Population and Their Relation with Bone Mineral Density: The Rotterdam Study. Eur. J. Nutr..

[41] Barbosa De Aguiar O., Godoi A.G., Ii V., Dias P.L., Iii B., Barbosa O., Rua A. (2019). The Identification of Food Patterns: A Comparison of Principal Component and Principal Axis Factoring Techniques. Rev. Bras. Epidemiol..

[42] González-Toribio J., Hunot-Alexander C., Vásquez-Garibay E.M., Larrosa-Haro A., Casillas-Toral E., Curiel-Curiel C.P. (2023). Association between Maternal and Toddler Appetitive Traits in a Mexican Population. Behav. Sci..

[43] Herle M., Fildes A., Llewellyn C.H. (2018). Emotional Eating Is Learned Not Inherited in Children, Regardless of Obesity Risk. Pediatr. Obes..

[44] Finnane J.M., Jansen E., Mallan K.M., Daniels L.A. (2017). Mealtime Structure and Responsive Feeding Practices Are Associated With Less Food Fussiness and More Food Enjoyment in Children. J. Nutr. Educ. Behav..

[45] Pickard A., Croker H., Farrow C., Haycraft E., Herle M., Kininmonth A., Llewellyn C., Blissett J. (2022). Appetite in Preschoolers: Producing Evidence for Tailoring Interventions Effectively—The APPETItE Study Protocol. Appetite.

[46] Miller V., Micha R., Choi E., Karageorgou D., Webb P., Mozaffarian D. (2022). Evaluation of the Quality of Evidence of the Association of Foods and Nutrients with Cardiovascular Disease and Diabetes: A Systematic Review. JAMA Netw. Open.

[47] Marrón-Ponce J.A., Sánchez-Pimienta T.G., Rodríguez-Ramírez S., Batis C., Cediel G. (2023). Ultra-Processed Foods Consumption Reduces Dietary Diversity and Micronutrient Intake in the Mexican Population. J. Human Nutr. Diet..

[48] Prabhakaran D., Anand S., Gaziano T.A., Mbanya J.-C., Wu Y., Nugent R. (2017). Cardiovascular, Respiratory, and Related Disorders. Lancet.

[49] Monda A., de Stefano M.I., Villano I., Allocca S., Casillo M., Messina A., Monda V., Moscatelli F., Dipace A., Limone P. (2024). Ultra-Processed Food Intake and Increased Risk of Obesity: A Narrative Review. Foods.

[50] The Nutrition Source Kid’s Healthy Eating Plate—The Nutrition Source. https://nutritionsource.hsph.harvard.edu/kids-healthy-eating-plate/.

[51] Lichtenstein A.H., Appel L.J., Vadiveloo M., Hu F.B., Kris-Etherton P.M., Rebholz C.M., Sacks F.M., Thorndike A.N., Van Horn L., Wylie-Rosett J. (2021). 2021 Dietary Guidance to Improve Cardiovascular Health: A Scientific Statement from the American Heart Association. Circulation.

[52] Cena H., Calder P.C. (2020). Defining a Healthy Diet: Evidence for the Role of Contemporary Dietary Patterns in Health and Disease. Nutrients.

[53] Hidaka B.H., Kerling E.H., Thodosoff J.M., Sullivan D.K., Colombo J., Carlson S.E. (2016). Dietary Patterns of Early Childhood and Maternal Socioeconomic Status in a Unique Prospective Sample from a Randomized Controlled Trial of Prenatal DHA Supplementation. BMC Pediatr..

[54] Dalrymple K.V., Flynn A.C., Seed P.T., Briley A.L., O’Keeffe M., Godfrey K.M., Poston L. (2020). Associations between Dietary Patterns, Eating Behaviours, and Body Composition and Adiposity in 3-Year-Old Children of Mothers with Obesity. Pediatr. Obes..

[55] Popkin B.M., Reardon T. (2018). Obesity and the Food System Transformation in Latin America. Obes. Rev..

[56] Rivera-Dommarco J.A., Sánchez-Pimienta T.G., García-Guerra A., Ávila M.A., Cuevas Nasu L., Barquera S., Shamah-Levy T. Situación Nutricional de La Población En México Durante Los Últimos 120 Años. https://www.insp.mx/resources/images/stories/2023/docs/230127_Situacion%20_nutricional_dela_ooblacion_Mexico.pdf.

[57] Rauber F., Campagnolo P.D.B., Hoffman D.J., Vitolo M.R. (2015). Consumption of Ultra-Processed Food Products and Its Effects on Children’s Lipid Profiles: A Longitudinal Study. Nutr. Metab. Cardiovasc. Dis..

[58] Syrad H., Johnson L., Wardle J., Llewellyn C.H. (2015). Appetitive Traits and Food Intake Patterns in Early Life. Am. J. Clin. Nutr..

[59] Cole N.C., An R., Lee S.Y., Donovan S.M. (2017). Correlates of Picky Eating and Food Neophobia in Young Children: A Systematic Review and Meta-Analysis. Nutr. Rev..

[60] Redsell S.A., Slater V., Rose J., Olander E.K., Matvienko-Sikar K. (2021). Barriers and Enablers to Caregivers’ Responsive Feeding Behaviour: A Systematic Review to Inform Childhood Obesity Prevention. Obes. Rev..

[61] Black M.M., Aboud F.E. (2011). Responsive Feeding Is Embedded in a Theoretical Framework of Responsive Parenting. J. Nutr..

[62] Kininmonth A., Smith A., Carnell S., Steinsbekk S., Fildes A., Llewellyn C. (2021). The Association between Childhood Adiposity and Appetite Assessed Using the Child Eating Behavior Questionnaire and Baby Eating Behavior Questionnaire: A Systematic Review and Meta-Analysis. Obes. Rev..

[63] Schultink J.M., de Vries J.H.M., de Wild V.W.T., van Vliet M.S., van der Veek S.M.C., Martens V.E., de Graaf C., Jager G. (2021). Eating in the absence of hunger in 18-month-old children in a home setting. Pediatr. Obes..

[64] Chaidez V., Townsend M., Kaiser L.L. (2011). Toddler-Feeding Practices among Mexican American Mothers. A Qualitative Study. Appetite.

[65] Smith A.D., Herle M., Fildes A., Cooke L., Steinsbekk S., Llewellyn C.H. (2017). Food fussiness and food neophobia share a common etiology in early childhood. J. Child Psychol. Psychiatry.

[66] Dubois L., Bédard B., Goulet D., Prud’homme D., Tremblay R.E., Boivin M. (2022). Eating Behaviors, Dietary Patterns and Weight Status in Emerging Adulthood and Longitudinal Associations with Eating Behaviors in Early Childhood. Int. J. Behav. Nutr. Phys. Act..

[67] Manterola C., Otzen T. (2015). Los Sesgos En Investigación Clínica. Int. J. Morphol..

[68] Russell A., Jansen E., Burnett A.J., Lee J., Russell C.G. (2023). Children’s Eating Behaviours and Related Constructs: Conceptual and Theoretical Foundations and Their Implications. Int. J. Behav. Nutr. Phys. Act..

[69] Mallan K., Miller N., Henry C.J., Nicklas T.A., Nicklaus S. (2019). Effect of Parental Feeding Practices (i.e., Responsive Feeding) on Children’s Eating Behavior. Nurturing a Healthy Generation of Children: Research Gaps and Opportunities.

[70] Hennessy E., Hughes S.O., Goldberg J.P., Hyatt R.R., Economos C.D. (2012). Permissive Parental Feeding Behavior Is Associated with an Increase in Intake of Low-Nutrient-Dense Foods among American Children Living in Rural Communities. J. Acad. Nutr. Diet..

[71] Kerzner B., Milano K., Maclean W.C., Berall G., Stuart S., Chatoor I. (2015). A Practical Approach to Classifying and Managing Feeding Difficulties. Pediatrics.

