# Supplementary material for: Appetitive Traits and Dietary Patterns in Mexican Children Aged 12 to 36 Months
Source: Nutrients. 2025 May 27;17(11):1814. doi: 10.3390/nu17111814 (PMC12157002; doi:10.3390/nu17111814)
Supplement: Supplementary file 1 [file nutrients-17-01814-s001.zip › nutrients-3647960-supplementary.pdf]

Article

Appetitive traits and dietary patterns in Mexican children aged 12 to 36 Months

Astrid S Gil-Barrera, Claudia Hunot-Alexander, Clío Chávez-Palencia, Jocelyn González-Toribio *, Erika Casillas-Toral, D Citlalli Álvarez-Zaragoza, Alfredo Larrosa-Haro, Edgar Vásquez-Garibay.

Table S1: Odds ratio analyses of dietary pattern adherence by appetitive trait category

Appetitive Trait		Dietary Pattern		Odds Ratio (95% CI)	p-value
	High/Low Values		Yes/No Adherence		
Food Responsiveness	High = 16 (31.4%)<br>Low = 35 (68.6%)	Processed	Yes = 16 (36.4%)<br>No = 28 (63.6%)	2.2 (1.1–4.5)	0.026
	High = 24 (17.8%)<br>Low = 111 (82.2%)	Healthy	Yes = 24 (21.6%),<br>No = 87 (78.4%)	0.7 (0.4–1.3)	0.228
	High = 4 (13.3%)<br>Low = 26 (86.7%)	Transition/ Modern Mexican	Yes = 4 (15.4%)<br>No = 22 (84.6%)	0.5 (0.2–1.5)	0.209
Emotional Overeating	High = 3 (5.5%)<br>Low = 52 (94.5%))	Processed	Yes = 3 (5.8%)<br>No = 49 (94.2%)	0.9 (0.2–3.7)	0.991
	High = 8 (5.5%)<br>Low = 137 (94.5%)	Healthy	Yes = 8 (5.8%)<br>No = 129 (94.2%)	0.9 (0.3–3.0)	0.991
	High = 2 (6.1%)<br>Low = 31 (93.9%)	Transition/ Modern Mexican	Yes = 2 (6.5%)<br>No = 29 (93.5%)	1.1 (0.2–5.6)	0.991
Enjoyment of Food	High = 52 (96.3%)<br>Low = 2 (3.7%)	Processed	Yes = 52 (25.2%)<br>No = 154 (74.8%)	1.0 (0.2–4.6)	0.969
	High = 141 (97.2%)<br>Low = 4 (2.8%)	Healthy	Yes = 141 (64.1%)<br>No = 79 (35.9%)	0.4 (0.1–1.5)	0.188
	High = 28 (90.3%)<br>Low = 3 (9.7%)	Transition/ Modern Mexican	Yes = 28 (53.8%)<br>No = 24 (46.2%)	3.4 (0.7–16.5)	0.118
Satiety Responsiveness	High = 13 (24.5%)<br>Low = 40 (75.5%)	Processed	Yes = 13 (22.4%)<br>No = 45 (77.6%)	0.9 (0.4–1.9)	0.799
	High = 34 (24.6%)<br>Low = 104 (75.4%)	Healthy	Yes = 34 (33.0%)<br>No = 69 (67.0%)	0.8 (0.4–1.4)	0.410
	High = 11 (20.0%)<br>Low = 19 (80.0%)	Transition/ Modern Mexican	Yes = 11 (29.7%)<br>No = 26 (70.3%)	1.7 (0.7–4.1)	0.196
Food Fussiness	High = 11 (20.0%)<br>Low = 44 (80.0%)	Processed	Yes = 11 (20.0%)<br>No = 44 (80.0%)	0.9 (0.4–2.2)	0.802
	High = 21 (14.5%)<br>Low = 124 (85.5%)	Healthy	Yes = 21 (22.3%)<br>No = 73 (77.7%)	0.4 (0.2–0.8)	0.003
	High = 16 (50.0%)<br>Low = 16 (50.0%)	Transition/ Modern Mexican	Yes = 16 (33.3%)<br>No = 32 (66.7%)	5.2 (2.4–11.6)	<0.001
Slowness in Eating	High = 13 (23.2%)<br>Low = 43 (76.8%)	Processed	Yes = 13 (21.7%)<br>No = 47 (78.3%)	0.8 (0.4–1.9)	0.787
	High = 35 (26.1%)<br>Low = 99 (73.9%)	Healthy	Yes = 35 (35.4%)<br>No = 64 (64.6%)	1.0 (0.6–1.7)	0.733
	High = 11 (39.3%)	Transition/Moder	Yes = 11 (32.4%)	1.9 (0.8–4.4)	0.123

Low = 17 (60.7%)	n Mexican	No = 23 (67.6%)

Note: Some odds ratios were calculated from groups with small cell sizes (fewer than five subjects), which may limit the reliability and generalizability of the estimates. These results should be interpreted with caution and considered exploratory, providing preliminary insight for future research.
